# *α*TAT1 downregulation induces mitotic catastrophe in HeLa and A549 cells

**DOI:** 10.1038/cddiscovery.2016.6

**Published:** 2016-02-15

**Authors:** J-Y Chien, S-D Tsen, C-C Chien, H-W Liu, C-Y Tung, C-H Lin

**Affiliations:** 1 Institute of Microbiology and Immunology, National Yang-Ming University, Taipei, Taiwan; 2 VYM Genome Research Center, National Yang-Ming University, Taipei, Taiwan; 3 Institute of Biophotonics, National Yang-Ming University, Taipei, Taiwan; 4 Institute of Clinical Medicine, National Yang-Ming University, Taipei, Taiwan

## Abstract

*α*-Tubulin acetyltransferase 1 (*α*TAT1) controls reversible acetylation on Lys40 of *α*-tubulin and modulates multiple cellular functions. *α*TAT1 depletion induced morphological defects of touch receptor neurons in *Caenorhabditis elegans* and impaired cell adhesion and contact inhibition in mouse embryonic fibroblasts, however, no morphological or proliferation defects in human RPE-hTERT cells were found after *α*TAT1-specific siRNA treatment. Here, we compared the effect of three *α*TAT1-specific shRNAs on proliferation and morphology in two human cell lines, HeLa and A549. The more efficient two shRNAs induced mitotic catastrophe in both cell lines and the most efficient one also decreased F-actin and focal adhesions. Further analysis revealed that *α*TAT1 downregulation increased *γ*-H2AX, but not other DNA damage markers p-CHK1 and p-CHK2, along with marginal change in microtubule outgrowth speed and inter-kinetochore distance. Overexpression of *α*TAT1 could not precisely mimic the distribution and concentration of endogenous acetylated *α*-tubulin (Ac-Tu), although no overt phenotype change was observed, meanwhile, this could not completely prevent *α*TAT1 downregulation-induced deficiencies. We therefore conclude that efficient *α*TAT1 downregulation could impair actin architecture and induce mitotic catastrophe in HeLa and A549 cells through mechanisms partly independent of Ac-Tu.

## Introduction

Post-translational modifications of a subset of tubulin are involved in regulating cellular functions. Most modifications are near the C terminus of *α*-tubulin, which extends to the outside environment after being constituted in the microtubule; an exception is the reversible acetylation on Lys40.^1–3^ This modification was found to be enriched in stable or long-lived microtubules, such as axonemes. During cell division, kinetochore microtubule is acetylated on Lys40 after its assembly until midbody formation. It is conserved in a wide range of species, from the unicellular *Tetrahymena* to mammals. Only rare cells, such as PtK2, were found to be devoid of it.^4,5^

MEC-17 in *Caenorhabditis elegans* (*C. elegans*) and its homolog *α*TAT1 in other organisms are the major *α*-tubulin acetyltransferase; besides its auto acetylation, *α*-tubulin is the major substrate found to date.^6–8^ Depletion of MEC-17 or *α*TAT1 in *Tetrahymena*, *C. elegans*, and mice did not noticeably affect their growth; detailed analysis showed morphological defects of touch receptor neurons and touch insensitivity in *C. elegans* but only subtle changes in brain and testis organization were found in mice.^6,7,9–13^ In cell models, Ac-Tu was reported to be involved in microtubule dynamics,^8^ cell migration,^14–16^ motor protein transport,^17,18^ and DNA repair.^19^ However, *in vitro* experiments did not totally support the direct influence of *α*TAT1 or Ac-Tu on microtubule structure,^20^ dynamics,^20,21^ or motor protein transport.^22^ On the other hand, *α*TAT1 depletion-induced phenotypes can be partially restored by its enzymatically inactive mutant,^8,11^ implying it can play other roles independent of the acetylation activity.

Previous study in human RPE-hTERT cells did not find morphological or proliferation defect after *α*TAT1 depletion,^7^ however, *ex vivo* cultured *αTat1* ^−/−^ mouse embryonic fibroblasts displayed impaired cell adhesion and contact inhibition.^23^ To verify whether these results were due to different roles of *α*TAT1 in different cell types, we compared the effect of three *α*TAT1-specific shRNAs in another two human cell lines, HeLa and A549. Our results showed that actin architecture and cell cycle progression could be affected after efficient *α*TAT1 downregulation and Ac-Tu is not the only player through which *α*TAT1 exerts its effect.

## Results

### *α*TAT1 downregulation did not disrupt mitotic spindle formation

**Figure 1:**
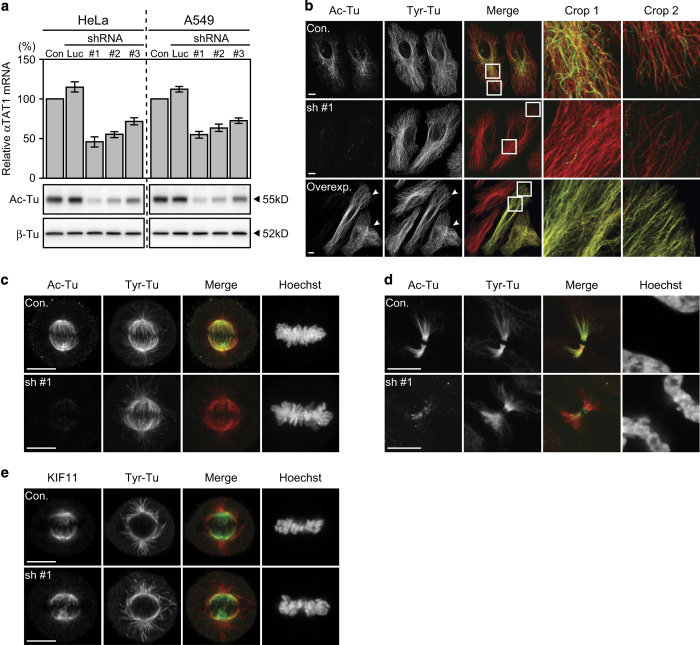
Efficiency of tested shRNAs and their effect on Ac-Tu. (**a**) *α*TAT1 mRNA and Ac-Tu levels were determined by real-time PCR and western blotting, respectively. The *α*TAT1/GAPDH mRNA ratio of the control group was set as 100% in each repeat; data shown in mean±S.D. (*N*=3). (**b**–**d**) Confocal images of HeLa cells immunostained with antibodies against Ac-Tu (green) and tyrosylated *α*-tubulin (Tyr-Tu, red). (**b**) Subcellular regions close to the nucleus (Crop 1) and cell periphery (Crop 2) are enlarged. Arrowheads indicate cells stably expressing *α*TAT1; their Ac-Tu intensity was so high that Ac-Tu in control cells was barely visualized under this image capture setting. (**e**) Confocal images of metaphase HeLa cells immunostained with anti-KIF11 antibody. Single optical section of the middle plane is shown here. Scale bar, 10 *μ*m.

We introduced *α*TAT1-specific shRNA corresponding to the published siRNA sequence^7^ (sh #3 in this study) and another two *α*TAT1-specific shRNAs (sh #1 and sh #2) into HeLa and A549 cells by the lentiviral system, which provides a more stable downregulation effect over the experimental period. Experiments were carried out during 4–12 days post transduction to avoid decay of downregulation effect. The residual *α*TAT1 mRNA and Ac-Tu level were determined by real-time PCR and western blotting, respectively. Results showed that all three shRNAs effectively downregulated *α*TAT1, while sh #1 had the highest efficiency and sh #3 the least (Figure 1a).

In control cells, Ac-Tu was more enriched in curved microtubule near the microtubule-organizing center than in the cell periphery; after *α*TAT1 downregulation, only weak fragments or punctate Ac-Tu staining were observed (Figure 1b). In contrast, stable expression of *α*TAT1 dramatically increased Ac-Tu all over the cell even transduced at low effective multiplicity of infection and cells were stabilized over 4 weeks (Figure 1b); nevertheless, cell morphology, growth, and cell cycle distribution (Figures 7a–c and data not shown) were not noticeably changed. During M phase, kinetochore microtubule was highly acetylated until midbody formation, but mitotic spindle was in general normally shaped without this modification and chromosome alignment at metaphase was not noticeably altered (Figures 1c and d). The distribution of KIF11 (also known as Eg5), a kinesin-like protein required for mitotic spindle formation and stability,^24^ was still associated with the kinetochore microtubule after *α*TAT1 downregulation (Figure 1e).

### *α*TAT1 downregulation decreased F-actin and altered *α*-adaptin distribution

**Figure 2:**
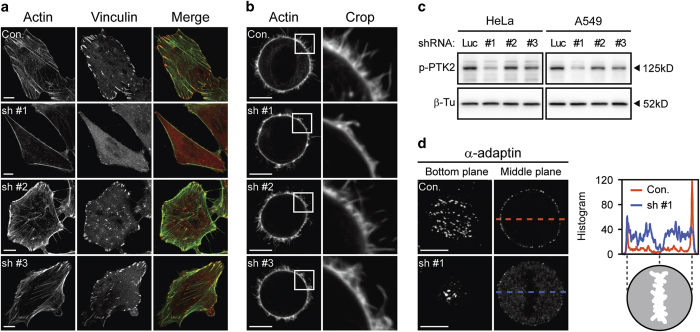
*α*TAT1 downregulation decreased F-actin and altered *α*-adaptin distribution. (**a**) Confocal images of A549 cells visualized with Alexa Fluor 488 phalloidin (green) and immunostained with anti-vinculin antibody (red). (**b**) Confocal images of metaphase HeLa cells visualized with Alexa Fluor 488 phalloidin. Single optical section of the middle plane is shown. (**c**) pTyr397-PTK2 level determined by western blotting. The same protein extract was used in Figures 1a and 2c, therefore the same blotting of *β*-Tu is presented in both figures. (**d**) Confocal images of metaphase HeLa cells immunostained with anti-*α*-adaptin antibody. Single optical section of the bottom or middle planes are shown. The signal intensity across the dashed lines is plotted in the right panel and the cartoon below indicates the relative position of the metaphase cell. Scale bar, 10 *μ*m.

We next examined the actin architecture. Fluorescent-labeled phalloidin and immunostaining with anti-vinculin antibody revealed significant decrease in F-actin and focal adhesions after sh #1 treatment (Figure 2a), which was consistent with the phenomena observed in the *αTat1*^−/−^ mouse embryonic fibroblasts.^23^ Meanwhile, decreased actin-rich protrusions at metaphase were also observed (Figure 2b). Less efficient sh #2 or sh #3 had subtle effect in this aspect and western blotting also revealed that sh #1 decreased pTyr397-PTK2 (also known as FAK) more intensively than sh #2 or sh #3 did (Figure 2c). Distribution of a previously reported *α*TAT1-associated protein *α*-adaptin^16^ was also affected (Figure 2d). Immunofluorescence staining revealed dozens of foci at the bottom plane of control metaphase cells, while the number and spanning area of foci were decreased after sh #1 treatment. At the middle plane, control cells showed punctate staining on the cell membrane with dark cytosolic background, whereas sh #1 treatment resulted in diffused cytosolic signals with fewer membrane spots.

### *α*TAT1 downregulation impaired cell growth with increased 4N and multiploid cells

**Figure 3:**
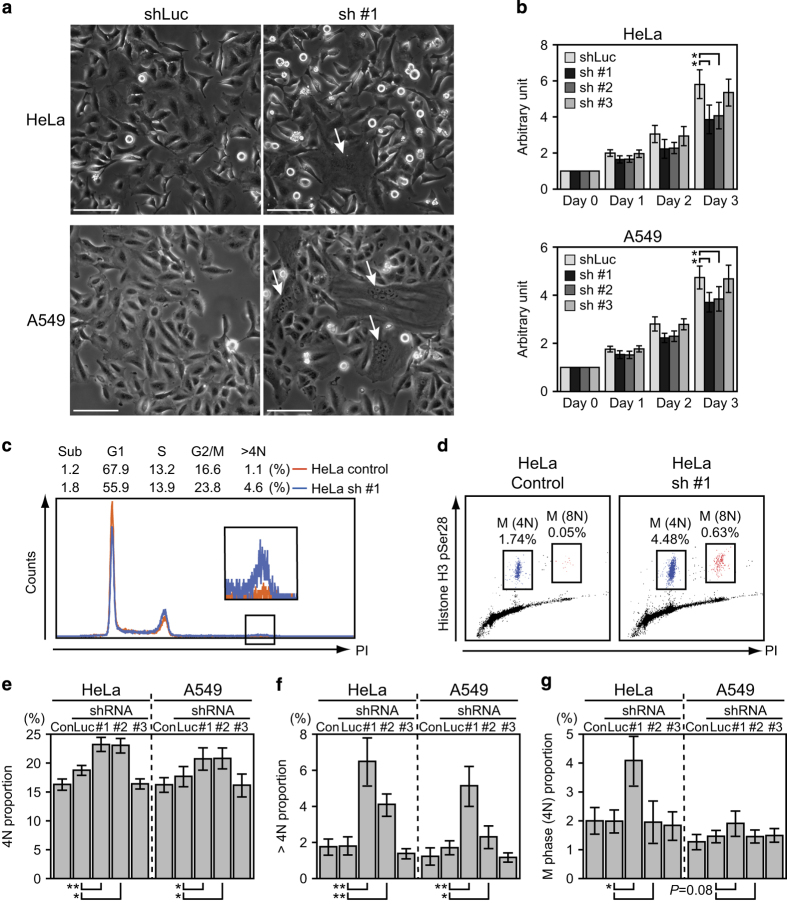
*α*TAT1 downregulation impaired cell growth, and increased 4N and multiploid population. (**a**) Phase contrast images of live cells showing increased rounded and abnormally large (arrows) cells after sh #1 treatment. Sacle bar, 100 *μ*m. (**b**) MTT assay of cell growth. In each treatment, the measurement of day 0 was set as 1 in the arbitrary unit and the fold change of day 1–3 to its day 0 is displayed; data shown in mean±S.D. (*N*=3). (**c** and **d**) Representative flowcytometry results of cell cycle distribution using propidium iodide to reveal the DNA content and histone H3 pSer28 as the M-phase marker. (**e**–**g**) Statistical results of flowcytometry data; 30 000 cells were analyzed in each sample and data shown in mean±S.D. (*N*=4). Proportion of 4N and histone H3 pSer28 positive cells are displayed in **g**. *P<0.05, **P<0.01.

Increased rounded, detached, and abnormally large sized cells were observed after 72 h of sh #1 and sh #2, but not sh #3 treatment in both cell lines (Figure 3a). MTT assay also revealed that sh #1 and sh #2 impaired cell growth (Figure 3b). The effect of sh #3 was comparable to control shRNA, which was consistent with the previous study performed in REP-hTRET cells.^7^ Analyzing their cell cycle distribution using flowcytometry with propidium iodide staining revealed that only sh #1 and sh #2 increased the 4N as well as multiploid cell population (Figures 3c, e and f). In addition, histone H3 pSer28 staining showed that sh #1 also increased the M-phase population but not sh #2 and HeLa was more susceptible than A549 in this aspect (Figures 3d and g), implying that the increased 4N cells by sh #1 were a combination of normal G2/M and tetraploid G1-phase cells while the increase seen after sh #2 treatment was mostly from normal G2 and tetraploid G1-phase cells.

### *α*TAT1 downregulation-induced mitotic catastrophe

**Figure 4:**
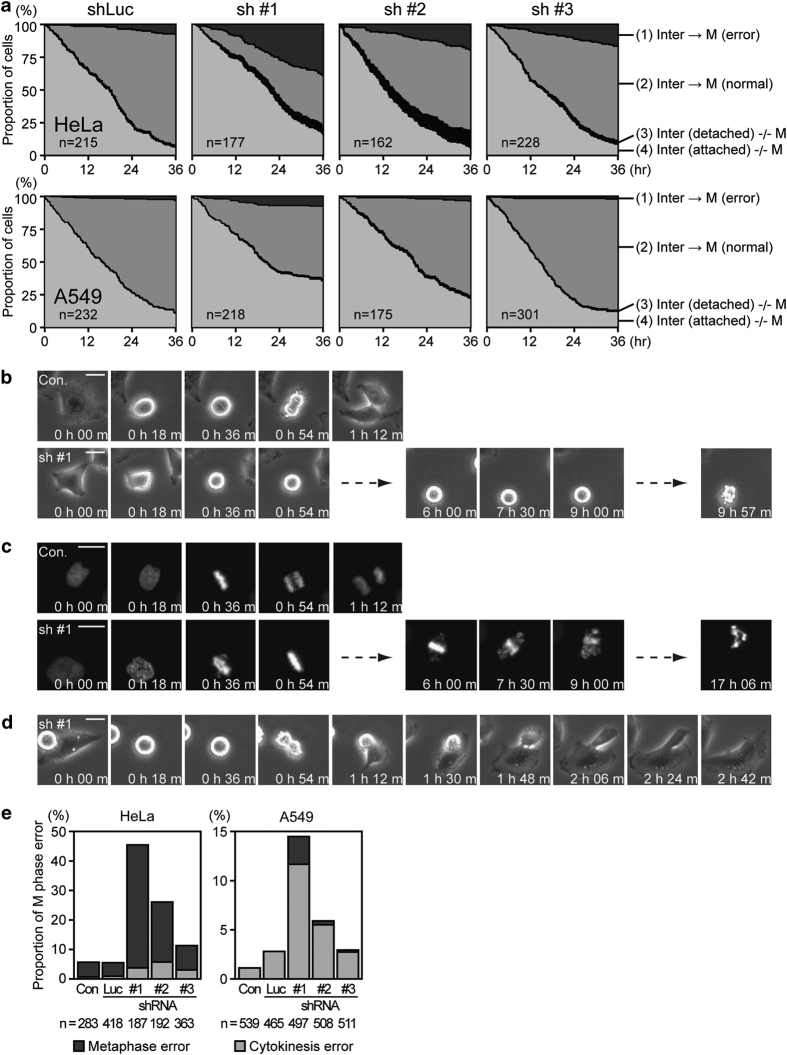
*α*TAT1 downregulation-induced mitotic catastrophe. (**a**) Cell fate until finishing the first round M phase in time-lapse recordings was traced manually and divided into four groups: (1) entered M phase and error at metaphase or cytokinesis was observed, (2) entered M phase and produced two daughter cells, (3) detached before entering M phase, and (4) attached not yet entering M phase. Time of the anaphase onset was used to represent M-phase time point. For each treatment, data from three independent experiments are pooled and the total cell number at the initial time point is presented on the graphs. (**b**–**d**) Representative phase contrast or fluorescence time serial images of sh #1-induced metaphase or cytokinesis error in HeLa cells. (**c**) Chromosomes visualized by stably expressing histone H2B-strawberry. (**e**) Statistical analysis of *α*TAT1 downregulation-induced M phase error. Only cells entering M phase are included in **e**; metaphase error indicates cells did not move on to anaphase, cytokinesis error indicates cells passed metaphase properly but did not produce two daughter cells. For each treatment, data from three independent experiments are pooled and the total M-phase events recorded are presented on the graphs. Scale bar, 25 *μ*m.

To further understand the fate of deficient cells, we monitored cell growth using time-lapse microscopy. During 36 h observation, cells detached at the interphase or stayed arrested without entering M phase were increased after sh #1 and sh #2 treatment (Figure 4a). For those entering M phase, 93.5% of the control shRNA-treated HeLa cells entered anaphase properly, mostly at about 1 h after starting to round up, whereas only 55.1% and 74.0% of sh #1- and sh #2-treated cells, respectively, did so (Figure 4e). Similar tendency was observed in A549 cells; although 97.2% of sh #1-treated cells still entered anaphase, this reduction was significant in comparison with 100.0% of observed control cells (*n*=539) and control shRNA-treated cells (*n*=465) did so (Figure 4e). Cells arrested at metaphase usually stayed rounded for several hours before their eventual detachment (Figure 4b). To verify whether chromosome alignment at metaphase was impaired after *α*TAT1 downregulation, HeLa cells stably expressing histone H2B-strawberry were used. Results revealed that chromosomes could be aligned at the metaphase plate of deficient cells, however, after sustaining several hours without entering anaphase, more and more chromosomes moved outward followed by cell detachment (Figure 4c). As for the sh #1- and sh #2-treated cells moving on to cytokinesis, the furrow ingression stage was not noticeably affected but 3.7% and 5.7% of HeLa and 11.7% and 5.5% of A549 cells still underwent furrow regression that mostly produced multiploid cells (Figures 4d and e). These characteristics were consistent with mitotic catastrophe, although HeLa and A549 cells showed slightly different susceptibility during cell cycle stages.

### *α*TAT1 downregulation increased *γ*-H2AX but not p-CHK1 or p-CHK2

**Figure 5:**
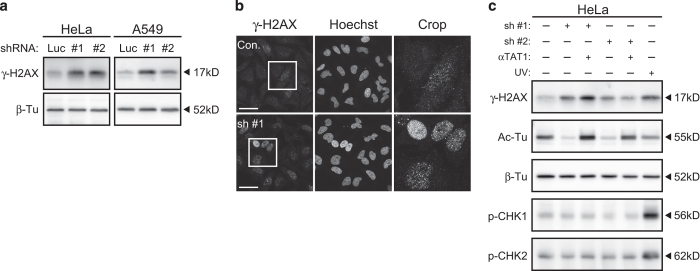
*α*TAT1 downregulation increased *γ*-H2AX but not p-CHK1 or p-CHK2. (**a**) *γ*-H2AX level determined by western blotting. (**b**) Confocal images of HeLa cells immunostained with anti-*γ*-H2AX antibody. (**c**) HeLa control or *α*TAT1 stably expressing cells were analyzed by western blotting after sh #1 or sh #2 treatment using antibodies against Ac-Tu, *γ*-H2AX, pSer345-CHK1, and pThr68-CHK2. Control cells exposed to UV irradiation then recovered for 4 h were used as a positive control. Scale bar, 25 *μ*m.

Mitotic catastrophe could be triggered by agents impairing DNA integrity or microtubule stability,^25^ meanwhile, proper control of *α*TAT1 or Ac-Tu level were found to be important to DNA repair^19^ and microtubule dynamics.^8^ We first examined whether *α*TAT1 downregulation increased DNA damage. Accumulation of *γ*-H2AX, the Ser139 phosphorylated form of histone H2AX, at the DNA double-strand break site makes it a marker of DNA damage; although its increase in scenarios without inducing DNA damage have also been reported.^26^ Western blotting revealed that efficient *α*TAT1 downregulation increased *γ*-H2AX in both cell lines (Figure 5a); immunofluorescence staining of *γ*-H2AX also showed increased bright punctate staining in the nuclei as well as the proportion of cells with this pattern (Figure 5b). However, another two DNA damage markers pSer345-CHK1 and pThr68-CHK2^27,28^ were not increased simultaneously (Figure 5c).

### *α*TAT1 downregulation marginally affected microtubule dynamics

**Figure 6:**
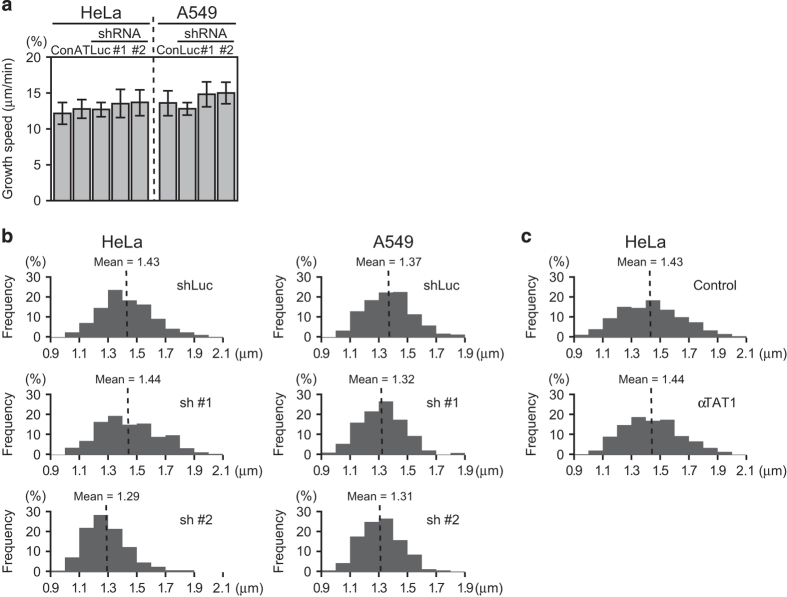
*α*TAT1 downregulation marginally affected microtubule dynamics. (**a**) EB3-GFP comets movement was analyzed using plusTipTracker software package to reveal microtubule growth speed. AT denotes *α*TAT1 stably expressing cells. In each treatment, 15 cells were analyzed; data shown in mean±S.D. (**b** and **c**) Distribution of inter-kinetochore distance. Cells were immunostained against an outer kinetochore protein NDC80 and Tyr-Tu. In each treatment, images of 15 cells were analyzed and results were pooled to obtain 400–600 sister kinetochore pairs.

We next examined whether *α*TAT1 downregulation affected microtubule outgrowth, which was reported to be decreased after *α*TAT1 shRNA treatment and increased after transfecting with YFP-*α*TAT1 in NIH 3T3 cells.^8^ Analysis of GFP-tagged end-binding protein 3 (EB3) comets movement showed that microtubule growth speed marginally increased after sh #1 or sh #2 treatment in both cell lines but was not significantly changed in HeLa cells stably expressing *α*TAT1 (Figure 6a). At metaphase, proper control of microtubule dynamics is required to establish optimal sister kinetochore tension and then for silencing the spindle assembly checkpoint. Immunostaining of an outer kinetochore protein NDC80 showed the distribution of inter-kinetochore distance was slightly downshifted after sh #2 treatment in both cell lines and sh #1 in A549 cells (Figure 6b). Unexpectedly, this tendency was not observed after sh #1 treatment in HeLa cells, in which the most M phase-arrested cells could be found. On the other hand, stably expressing *α*TAT1 did not noticeably alter the distribution of inter-kinetochore distance in HeLa cells (Figure 6c).

### *α*TAT1 overexpression maintained Ac-Tu level but did not completely prevent *α*TAT1 downregulation-induced deficiencies

**Figure 7:**
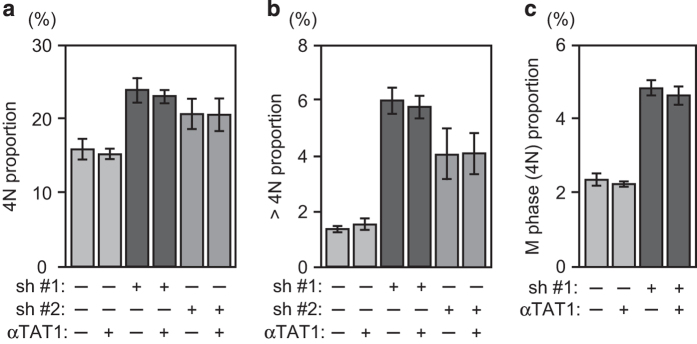
*α*TAT1 overexpression could not prevent sh #1- or sh #2-increased 4N and multiploid population. (**a**–**c**) HeLa control or *α*TAT1 stably expressing cells were analyzed by flowcytometry after sh #1 or sh #2 treatment using propidium iodide to reveal the DNA content and histone H3 pSer28 as the M-phase marker. In all, 30 000 cells were analyzed in each sample; data shown in mean±S.D. (*N*=4).

Ac-Tu was previously speculated to be the key player through which *α*TAT1 exerted its effect on microtubule, thereby modulating cellular functions. Latter studies proposed that *α*TAT1 also has other roles independent of its enzyme activity.^8,11^ To examine whether decreased Ac-Tu is responsible for the deficiencies observed here, HeLa cells stably expressing *α*TAT1 that could not be targeted by sh #1 and sh #2 were generated and then treated with these two shRNAs. Western blotting verified that Ac-Tu level was maintained after *α*TAT1 downregulation, and *γ*-H2AX increased by sh #2 but not sh #1 was partially reduced (Figure 5c). Further analysis of the cell cycle distribution revealed that 4N and multiploid population increased by sh #1 and sh #2 or M-phase population by sh #1 could not be prevented efficiently (Figures 7a–c), thus implying that metaphase and cytokinesis error could have occurred. Morphological change after sh #1 treatment was still observed (data not shown).

## Discussion

*α*TAT1 depletion was reported to impair cell adhesion and contact inhibition in mouse embryonic fibroblasts but not in human RPE-hTERT cells. In this study, we compared the effect of three *α*TAT1-specific shRNAs in another two human cell lines and found that actin architecture and cell cycle progression could be impaired after efficient *α*TAT1 downregulation. The most efficient sh #1 reduced F-actin and focal adhesions similar to the *αTat1*^−/−^ mouse embryonic fibroblasts and both sh #1 and sh #2 induced mitotic catastrophe. To our knowledge, this is the first report of shRNA-induced mitotic catastrophe in human cells.

The most intriguing questions raised in this study are why sh #3 almost did not affect cellular functions and stably expressing *α*TAT1 could not effectively prevent sh #1- and sh #2-induced deficiencies. The former question could be explained by the lower efficiency of sh #3 and the consequent higher residual Ac-Tu level than other two shRNAs. However, considering multiple *α*TAT1 transcription variants are expressed,^8,23^ we also suspect that individual shRNA could downregulate individual variant with different efficiencies. The hypothesis of specific *α*TAT1 variant is critical for specific cellular functions and could be more effectively downregulated by sh #1 but not sh #3 will be examined in our next study. On the basis of this hypothesis and the previously proved *α*TAT1 can exert its function independently of Ac-Tu, multiple *α*TAT1 variants could be required for different cellular functions instead of simply maintaining the Ac-Tu level. The fact that it is difficult to mimic the concentration and diversity of all variants may be the reason why *α*TAT1 overexpression did not completely rescue *α*TAT1 downregulation-induced deficiencies.

Accumulated DNA damage and microtubule instability are common reasons of mitotic catastrophe and both DNA repair and microtubule dynamics were reported to be modulated by *α*TAT1 or Ac-Tu level. We first examined a widely used DNA double-strand break marker *γ*-H2AX and found a significant increase after *α*TAT1 downregulation. However, its cell cycle-dependent increase and plateau at M phase were also reported.^26^ We then examined another two DNA damage markers, p-CHK1 and p-CHK2, and found that they were not increased. Thus implying that the increase in *γ*-H2AX after *α*TAT1 downregulation could be just a cell cycle-dependent manner caused by accumulated G2/M- and M-phase cells or DNA damages accumulated, but CHK1 and CHK2 were not phosphorylated. Further studies should be addressed to differentiate this.

*α*TAT1 depletion was also reported to decrease the microtubule plus-tip movement in NIH 3T3 cell.^8^ However, in HeLa and A549 cells, marginal increase of microtubule growth speed was found after *α*TAT1 downreglulation and stably expressing *α*TAT1 did not change this parameter in HeLa cells. Thus indicating microtubule outgrowth could not be simply modulated by Ac-Tu level in these two cell lines or other compensatory mechanisms raised shortly after *α*TAT1 shRNA treatment. We noticed that the fluorescence intensity of HeLa cells stably expressing GFP-*α*-tubulin was slightly increased after sh #1 treatment. Further studies should be addressed to verify whether endogenous tubulin is maintained at higher level after *α*TAT1 depletion and thus compensates microtubule polymerization.

We also examined whether sister kinetochore tension could be properly generated at metaphase after *α*TAT1 downreglulation. The distribution of inter-kinetochore distance was slightly downshifted after *α*TAT1 shRNA treatment except in the sh #1-treated HeLa group, in which the most increased M-phase population was found. Cells with relatively ideal mitotic spindle orientation and all NDC80 puncta located near the metaphase plate were imaged in this test. We were concerned that relatively normal cells would be preferentially selected which would decrease the difference, especially in the most affected sh #1-treated HeLa group. In time-lapse recordings, chromosomes of sh #1-treated HeLa cells failed to enter anaphase would be seen aligned at the metaphase plate during M phase. Thus we cannot rule out that *α*TAT1 could participate in other steps in the spindle assembly checkpoint.

On the other hand, sh #1 also impaired actin architecture and altered *α*-adaptin distribution. Actin dynamics is critical to mitotic spindle positioning and contractile ring formation during M phase. Meanwhile, depletion of *α*-adaptin was reported to perturb M-phase progression that increased multiploid cells,^29^ but the underlying mechanism is still not clear and further study should be done to see how this altered distribution by sh #1 contributes to inducing M-phase error. Besides, motor protein binding and its cargo transport were reported to be affected by Ac-Tu. The influence of *α*TAT1 depletion on vesicle trafficking to the midbody during cytokinesis should also be investigated in the future.

In conclusion, we found that efficient *α*TAT1 downregulation could impair human cellular functions. The impact could be different in different cell types, such as HeLa is more sensitive than A549 in metaphase arrest. Endogenous Ac-Tu level has recently been linked to metastatic behavior in breast cancer,^30^ therefore differentiating the role of *α*TAT1 in more types of cancer and its potential role as therapeutic target are worthy of further consideration.

## Materials and Methods

### Primary antibodies

Acetylated *α*-tubulin (Sigma-Aldrich, St. Louis, MO, USA, #T7451), tyrosylated *α*-tubulin (Genetex, Irvine, CA, USA, #GTX76511), *β*-tubulin (Sigma-Aldrich, #T5201), KIF11 (Abcam, Cambridge, MA, USA, #ab51976), vinculin (Sigma-Aldrich, #V9131), pTyr397-PTK2 (Thermo Fisher Scientific, Waltham, MA, USA #700255), *α*-adaptin (Thermo Fisher Scientific, #MA1-064), *γ*-H2AX (Genetex, #GTX127340; Cell Signaling Technology, Danvers, MA, USA, #9718), pSer345-CHK1 (Cell Signaling Technology, #2348), pThr68-CHK2 (Cell Signaling Technology, #2197), and NDC80 (Abcam, #ab3613).

### Cell culture and live cell imaging

HeLa and A549 cells were purchased from Bioresource Collection and Research Center of Taiwan (Hsinchu, Taiwan) and cultured in DMEM and F-12H containing 10% FBS, respectively. Cells were maintained at 37 °C in a humidified atmosphere of 5% CO2. For time-lapse recordings of cell proliferation, cells were seeded onto 3.5-cm glass bottom dishes (Alpha Plus, Taoyuan, Taiwan) at 5–10% confluency, allowed to attach overnight, and then observed with an inverted microscope equipped with ×10 or ×20 lenses, and a temperature- and CO_2_-controlled chamber; images were captured with 3-min intervals.

### RNA extraction, cDNA synthesis, and real-time PCR

For real-time PCR, total RNA was extracted using TRizol Reagent (Thermo Fisher Scientific) followed by reverse transcription using SuperScript III First-Strand Synthesis System (Thermo Fisher Scientific). mRNA level was quantified by TaqMan Real-Time PCR Assay (Thermo Fisher Scientific) using the probe Hs00227713_m1 against *α*TAT1 and GAPDH as the internal control.

### shRNA and plasmid DNA construction

shLuc (TRCN0000072243), sh #1 (TRCN0000263600), sh #2 (TRCN0000263597), and related vectors were obtained from National RNAi Core Facility Platform of Academia Sinica (Taipei, Taiwan). For sh #3 construction, sense oligonucleotide: 5′-CAACCGCCATGTTGTTTATATTCTCGAGAATATAAACAACATGGCGGTTTTTTTG-3′ and anti-sense oligonucleotide: 5′-AATTCAAAAAAACCGCCATGTTGTTTATATTCTCGAGAATATAAACAACATGGCGGTTGGTAC-3′ were synthesized, annealed, and inserted into the pLKO_TRC005 vector. *α*TAT1 cDNA was amplified from HeLa cDNA using the primers F: 5′-ATGGAGTTCCCGTTCGATG-3′ and R: 5′-TTAGTATCGACTCTCCTCAGAG-3′ and inserted into pLKO_AS2.EGFP or pLAS2w.Pbsd vectors. Histone H2B-strawberry obtained from Addgene was amplified and inserted into the pLKO_AS2.puro vector. EB3-GFP was provided by Chien-Yi Tung.

### Lentiviral delivery

Lentivirus production and transduction were performed following the supplier's instruction. Harvested virus-containing medium was filtered with 0.2 *μ*m filter disks, stored aliquots at −80 °C, and used within 3 months. Effective multiplicity of infection was determined by the percentage of transduced cells survived after 36 h drug treatment over non-treated cells. For shRNA and cDNA expression, 0.6–0.7 and 0.1–0.2 effective multiplicity of infection were applied, respectively. Experiments were performed during 4–12 days post shRNA transduction. Puromycin at 2.5 *μ*g/ml or blasticidin S at 5 *μ*g/ml were used when necessary.

### Western blotting

Cells were lysed with RIPA solution (20 mM Tris-HCl, pH 7.4, 150 mM NaCl, 1 mM EDTA, 1 mM EGTA, 1% NP-40, 1% sodium deoxycholate, and 1 mM Na_3_VO_4_) supplemented with protease inhibitor cocktail (Roche, Basel, Switzerland). Proteins separated by SDS-PAGE were transferred to PVDF membranes, blocked with 5% BSA in TBS-T (20 mM Tris-HCl, pH 7.4, 150 mM NaCl, and 0.1% Tween 20), and incubated with primary antibodies at 4 °C with agitation overnight. Membranes were then washed with TBS-T and incubated with horseradish peroxidase-conjugated secondary antibodies at room temperature for 1 h. After TBS-T wash, signals were visualized using enhanced chemiluminescence substrates.

### Immunofluorescence staining

After removing the culture medium and rinsed with PBS, cells were fixed with 3.7% formaldehyde at room temperature for 20 min. Cells were then permeabilized with 0.1% Triton X-100 prepared in PBS for 3 min, washed with PBS, and blocked in blocking solutions (1% BSA in PBS) for 30 min. Primary antibodies diluted at appropriate concentrations in blocking solution were then applied at room temperature for 1 h or at 4 °C overnight. After PBS wash, fluorophore-conjugated secondary antibodies in PBS were applied at room temperature for 1 h. Samples were observed using Leica SP5 (Leica, Wetzlar, Germany) or Zeiss LSM700 (Zeiss, Oberkochen, Germany) confocal microscopes.

### MTT assay

Three-thousand cells were seeded into each well of 96-well plates at eight replicates. At 12, 36, 60, and 84 h, medium was replaced with MTT reagent (900 *μ*l of culture medium with 100 *μ*l of MTT at 5 mg/ml in PBS) and incubated at 37 °C for 3 h. The solution was then replaced with 200 *μ*l of DMSO and incubated at 37 °C for 15 min before determining the absorbance at OD590 using a 620 nm reference filter.

### Flowcytometry

Cells were seeded onto 10 cm dishes at 15% confluency and medium was refreshed at 24 h to remove non-attached cells. Cells trypsinized at 48 h were resuspended with the original medium containing detached cells during 24–48 h and harvested by centrifugation. After PBS wash and centrifugation, cells were resuspended with PBS and ethanol was added drop by drop to a final concentration of 70%. Followed by fixation at −20 °C for 24–48 h, Alexa Fluor 488-conjugated histone H3 pSer28 antibody (BD, Franklin Lakes, NJ, USA, #558610) and propidium iodide were applied according to the manufacture’s instruction to reveal cell cycle distribution. Samples were analyzed with FACSCalibur (BD).

### Site-directed mutagenesis

QuikChange II site-directed mutagenesis kit (Agilent Technology, Santa Clara, CA, USA) was used to modify the expressed *α*TAT1 cDNA at the shRNA targeting region without changing the amino-acid sequence. First round of mutagenesis changed the targeting region of sh #1 and the product was used as a template in the second round to change the sh #2-targeting region. Primers used: F-sh #1: 5′-T GTG CAA CGC CAT GGC CAT GGA CGC GAG TTG TTT CAA TAC ATG TTG CAG AAG GAG CGA GTG-3′, R-sh #1: 5′-CAC TCG CTC CTT CTG CAA CAT GTA TTG AAA CAA CTC GCG TCC ATG GCC ATG GCG TTG CAC A-3′, F-sh #2: 5′-C AAG AAG CTC TTT GTA CTG GAC GAC CGA GAA GCC CAC AAC GAG GTA GAA CCA CTT TGC ATC-3′, and R-sh #2: 5′-GAT GCA AAG TGG TTC TAC CTC GTT GTG GGC TTC TCG GTC GTC CAG TAC AAA GAG CTT CTT G-3′.

### Microtubule dynamics

For microtubule outgrowth, cells were reverse transfected with 250 ng of EB3-GFP plasmid per 3.5-cm dish using T-Pro NRT II reagent (T-Pro Biotechnology, New Taipei City, Taiwan), allowed to attach overnight, and then observed with the same device as mentioned in the live cell imaging, except a ×100 objective lens with a lens heater were used and the top surface of lens was maintained at 37 °C; images were captured with 1 s intervals. Results were then analyzed with plusTipTracker software package.^31^ For inter-kinetochore distances, cells were immunostained with antibodies against NDC80 and Tyr-Tu to reveal the outer kinetochore position. Z-stack images of cells with all NDC80 puncta located near the metaphase plate were captured under a confocal microscope. The distance of unambiguous kinetochore pairs were measured in the ZEN software (Zeiss).

### Statistical analysis

All data are presented as means±S.D. Statistical significance between two data sets were compared by Student’s *t*-test.

## Abbreviations

*α*ΤΑΤ1: *α*-tubulin acetyltransferase 1
Ac-Tu: acetylated *α*-tubulin
Tyr-Tu: tyrosylated *α*-tubulin.
